# Challenges of fully online learning for dermatology education: a retrospective study

**DOI:** 10.3389/fmed.2023.1242772

**Published:** 2023-11-30

**Authors:** Yunfang Meng, Mingxia Sun, Jing Guo, Jing Jiao, Ningning Dang

**Affiliations:** ^1^Department of Dermatology, Shandong Provincial Hospital Affiliated to Shandong First Medical University, Jinan, Shandong, China; ^2^Department of Dermatology, Central Hospital Affiliated to Shandong First Medical University, Jinan, Shandong, China

**Keywords:** online learning, dermatology education, COVID-19 pandemic, live interaction, self-study ability

## Abstract

**Background:**

Blended learning has proven to be an effective teaching strategy. During the COVID-19 pandemic in 2019, educational institutions worldwide switched to online learning. However, there is limited research on the effectiveness of blended learning and fully online learning. This study aims to evaluate and compare whether pure online learning is as effective as traditional blended learning by taking the example of dermatology education.

**Methods:**

The researchers compared traditional blended learning and fully online learning by evaluating the achievement scores of undergraduate students in a dermatology course in the academic years 2019 and 2020, respectively, at the Shandong First Medical University, China. In 2019, students undertook small private online courses (SPOCs) combined with face-to-face teacher-led learning. In 2020, live teacher-led learning replaced face-to-face teacher-led learning. The researchers also conducted a questionnaire survey in 2020.

**Results:**

The scores of students in 2019 were significantly higher than in 2020 (*p* = 0.002). There was no significant difference in the distribution of achievement variance in the scores between the two academic years. In the questionnaire survey, the majority of the students rated highly the fully online education mode and responded that pure online learning enhanced their self-study ability.

**Conclusion:**

The present study shows that fully online learning currently does not perform as well as traditional blended learning in terms of examination scores due to some limitations. However, pure online education has several advantages over traditional blended education. Online courses should be improved to ignite students’ interest and increase their learning efficiency.

## 1 Introduction

Blended learning, also called hybrid learning, combines online learning with classroom teaching and has been proven to be an effective teaching strategy. With the universal availability of Wi-Fi and the increasing presence of online teaching platforms, blended learning has become a commonly used method for teaching (1, 2).

The outbreak of the COVID-19 pandemic at the end of 2019 brought a significant shift in teaching, with educational institutions worldwide switching to online learning. In China, the Shandong First Medical University also closed its campus due to lockdown regulations and transitioned from blended learning in dermatology to pure online learning, including self-learning with small private online courses (SPOCs) and live video platforms. SPOCs have gained global popularity in recent years and have emerged as an effective online educational program with simpler management procedures (3, 4).

Dermatology is a compulsory course for students majoring in clinical medicine. Many students find dermatology challenging because they need to learn about hundreds of diseases within a limited timeframe, and some struggle to keep up with classroom courses. Fortunately, the development of online learning has shown benefits for learning dermatology (4, 5). Students can review the lessons repeatedly and search for information while participating in online courses (6, 7). To enhance students’ education efficiency and develop their technical skills, the Shandong First Medical University introduced blended teaching for the dermatology course in 2019 and received positive feedback from students. Following the outbreak of the COVID-19 pandemic in 2019, the college began to reassess its dermatology teaching model and shifted from blended learning to pure online learning.

However, whether the transition to online learning is as effective as traditional blended learning remains unclear. This study aims to compare traditional blended learning and fully online learning by evaluating the achievement scores of dermatology students in the final examination and their perceptions of the different learning modes. The study findings will guide and encourage dermatology educators to adjust teaching methods to meet the requirements of the students in the multimedia era.

## 2 Materials and methods

**Figure 1:**
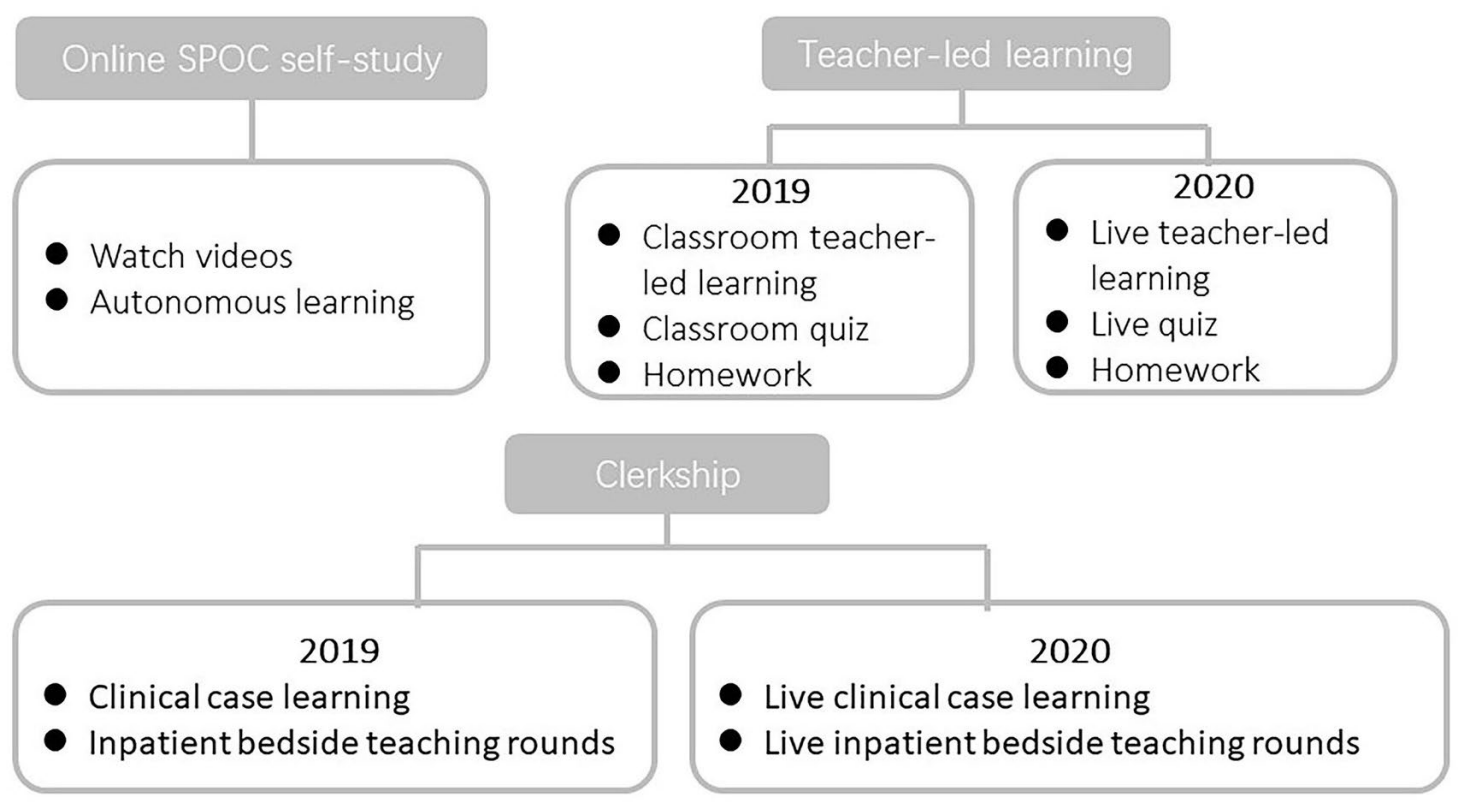
“Course design.” The course comprises 16 teaching hours for theory and 16 h for clerkship over one semester. For the theoretical component, dermatology was taught through a combination of small private online courses and traditional face-to-face teacher-led learning in 2019. In 2020, live teacher-led learning replaced face-to-face teacher-led learning. As for the clerkship part, in 2019, students primarily learned about diseases in the outpatient and inpatient departments through observation. In 2020, they learned about clinical cases online through live video streaming.

The Shandong First Medical University, China adopted the blended teaching mode for the dermatology course in 2019 (Figure 1). A high-quality SPOC was designed and implemented for online learning in combination with traditional face-to-face teaching. In 2020, following the outbreak of the COVID-19 pandemic, face-to-face teacher-led learning was suspended due to lockdown regulations, and live teacher-led learning was introduced. The clerkship courses were also shifted to live clinical case learning and live inpatient bedside teaching rounds. The curriculum comprised a total of 16 credit hours, with 8 credit hours allocated to 50 short videos related to the SPOC and another 8 credit hours for face-to-face classroom teaching or live online classes. A total of 84 and 113 students enrolled in dermatology courses in 2019 and 2020, respectively.

The SPOC videos for the dermatology course^1^ were designed by the Department of Dermatology of Shandong Provincial Hospital, affiliated with Shandong First Medical University. The videos received highly positive feedback from tens of thousands of learners (22,252 extramural MOOC learners and 754 internal SPOC learners). The microlesson videos used in the dermatology courses in this study were selected from SPOC and revised several times based on learner feedback. For the live teacher-led learning mode, the teachers and students discussed topics and issues together during scheduled real-time lessons.

All students took an examination and completed a questionnaire survey at the end of the course, both conducted online. The examination tested students’ clinical knowledge of dermatology, venereology, and allergy. The questionnaire was designed by the teaching faculty to evaluate students’ acceptance of the two teaching methods. The questionnaire survey included questions regarding students’ preferences for the live teacher-led learning mode and blended teaching mode, as well as the advantages and disadvantages of online learning.

Achievement scores in the final examinations in 2019 and 2020 were compared. The data from the questionnaire survey in 2020 were analyzed for this study. A total of 113 questionnaires were distributed in 2020, and the response rate was 100%. The Shapiro–Wilk method was used to test the quantitative data for normality, and a t-test was conducted to compare the normal data, which were expressed as mean ± standard deviation. A Mann–Whitney-Wilcoxon rank sum test was performed to compare the skewed data, expressed as median (p25, p75). The Chi-squared test was conducted to compare categorical data, presented as numbers (percentages). All statistical analyses were performed using SPSS version 13. The statistical significance level was set at *p* < 0.05. The baseline analysis compared the age and gender of students and did not find any statistical differences (*p* > 0.05).

The study was reviewed and approved by the Biomedical Research Ethical Committee of Shandong Provincial Hospital. Prior to distributing the questionnaire, the students were verbally informed about the study and provided their informed consent. The names of the students were anonymized to maintain confidentiality.

## 3 Results

**Figure 2:**
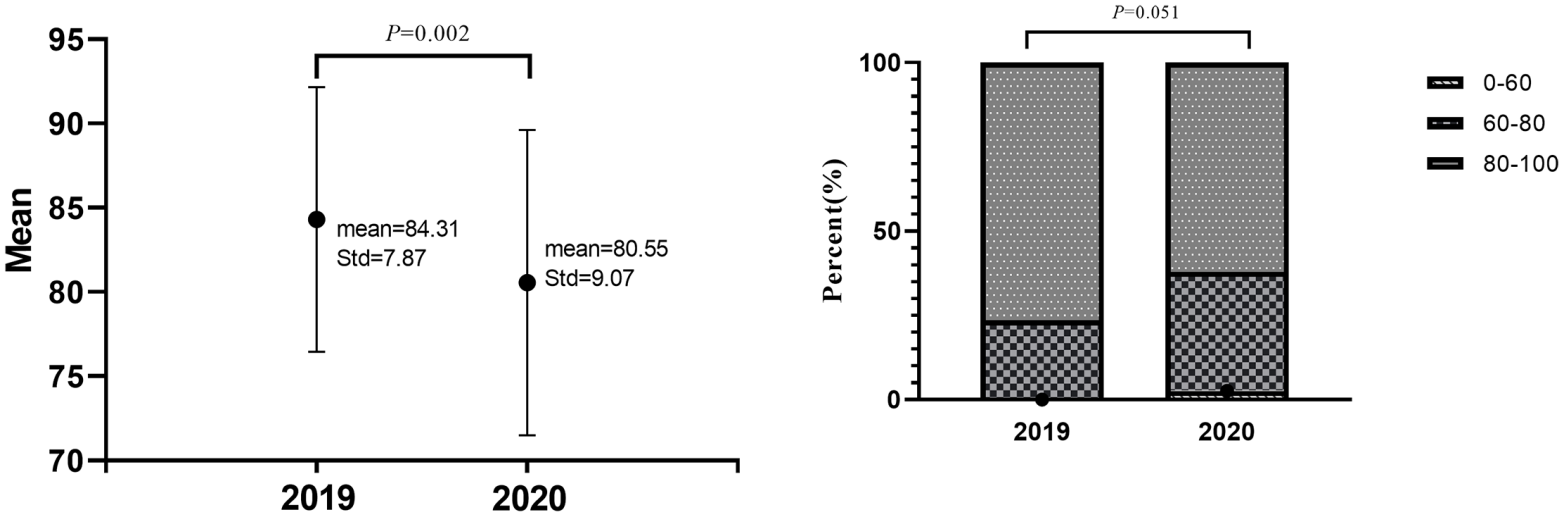
“Comparison of examination scores.” **(A)** The average scores for the academic years 2019 and 2020 were calculated. **(B)** The distribution of achievement variance at different levels for 2019 and 2020 was compared.

The average scores in academic years 2019 and 2020 were calculated. The average score in 2019 was 84.31, and in 2020, it was 80.55, with a statistically significant difference (*p* = 0.002) (Figure 2A). The highest score in 2019 was 96, and in 2020, it was 95 (Table 1). The distribution of achievement variance at different levels in 2019 and 2020 was compared. In 2019, no student scored below 60 points, 22.62% scored between 60 and 80 points, and 77.38% scored more than 80 points. In 2020, 2.65% of students scored below 60 points, 33.63% scored between 60 and 80 points, and 63.72% scored more than 80 points. There was no significant difference in the distribution of achievement variance between the two academic years (Figure 2B).

**Table 1:** Basic information about the students.

	2019	2020	Statistical value	*p*-value
Age (in years)	22 (20, 24)	22 (20, 24)	*Z* = −0.371	0.711
Sex	Male 32 (38.1%) Female 52 (61.9%)	Male 40 (38.1%) Female 52 (35.4%)	*χ*^2^ = 0.151	0.697

Online questionnaire surveys were conducted for students every year. The survey results for 2020 were analyzed in this study because fully online teaching was introduced that year. Most of the 113 students provided positive feedback about online teaching: 84.96% of the students favored the flexible learning schedule, 89.38% enjoyed repeated access to the learning content, and 67.26% felt relaxed while learning online. However, students also noted some disadvantages of online learning that cannot be ignored. Firstly, online learning is too dependent on a stable network connection for learning (53.10% of total students). Secondly, online learning often leads to poor learning motivation and low learning efficiency (46.90%). Thirdly, students could not interact with teachers and classmates intensively during online learning (41.59%). Lastly, the learning outcomes of fully online learning were not as good as that of traditional blended learning (27.43%), despite abundant learning tasks (10.62%) (Table 2).

**Table 2:** Advantages and disadvantages of online learning.

Questionnaire item	Agree (*n*[%])
*Advantages*	
The learning schedule is flexible.	96(84.96)
Students can learn the content on a repeated basis.	10(89.38)
Students feel more relaxed.	76(67.26)
*Disadvantages*	
Online learning is too dependent on a stable network for learning.	60(53.10)
Online learning often leads to poor learning motivation and low learning efficiency.	53(46.90)
Students could not interact with teachers and classmates intensively during online learning.	47(41.59)
The learning results of fully online learning were not as good as those of traditional blended learning.	31(27.43)
Online learning tasks are difficult.	12(10.62)

In the questionnaire survey, we placed extra emphasis on the optimization of teaching methods. When it comes to interaction methods, 84.07% of students preferred interactions during or immediately after live teacher-led learning through an online meeting application, 45.13% were inclined to communicate through SPOC discussion forums, while 27.43% preferred conducting discussions on WeChat. For online teaching methods, most students (78.76%) felt that a combination of online video self-study and live teacher-led learning worked best for them, while 11.50% of students preferred video self-study, and 9.73% liked live teacher-led learning only. The preference for offline clerkship courses was evident. Most students (69.14%) preferred offline clerkship courses, while 48.15% of students were satisfied with the online courses, and 49.38% thought that offline classroom teaching worked best for them. This result was not surprising, considering the visual nature of dermatology. Students preferred mostly PowerPoint presentations (95.58%), followed by course videos (91.15%), online homework (71.68%), e-textbooks (52.21%), related websites or books (45.13%), related literature (41.59%), and other learning sources (0.88%) (Table 3).

**Table 3:** Survey on the acceptance of different teaching modes.

Questionnaire item	Agree (*n*[%])
*What is your favorite interaction mode during online learning?*	
Interactions during or immediately after live teacher-led learning through an online meeting application.	95(84.07)
Communication through SPOC discussion forums.	51(45.13)
Discussion on WeChat.	31(27.43)
*What is your favorite online teaching mode?*	
Combination of online video self-study and live teacher-led learning.	89(78.76)
Video self-study only	13(11.50)
Live teacher-led learning only	11(9.73)
*Which learning mode works the best for you?*	
Face-to-face clerkship courses	78(69.14)
Online courses	54(48.15)
Face-to-face classroom teaching	56(49.38)
*What learning resources do you think should be provided for online learning?*	
Course PowerPoint presentations	108(95.58)
Course videos	103(91.15)
Online homework	81(71.68)
E-textbook	59(52.21)
Related websites or books	51(45.13)
Related literature	47(41.59)
Others	1(0.88)

Understanding and clarity of the concepts through online learning were also surveyed. The majority of the students (81.42%) responded that the subject was mostly mastered. A total of 5.31% of students fully mastered the concepts, and 12.39% of students partially mastered the subject. However, 0.88% of students only gained a limited understanding of the concepts. At the end of the semester, 79.65% of students felt that fully online learning enhanced their self-study ability, while 20.35% of students responded that they gained less from fully online learning than from classroom teacher-led learning. The satisfaction rate for fully online learning was as expected. About 54.87% of students in 2020 were satisfied with pure online learning, 38.94% scored it as excellent, while 6.19% considered it an average mode of learning (Table 4).

**Table 4:** Student assessment.

Questionnaire item	Agree (*n*[%])
*What is your mastery over the subject?*	
Fully mastered	6(5.31)
Mostly mastered	92(81.42)
Partially mastered	14(12.39)
A little mastered	1(0.88)
*What did you get from this online course?*	
Fully online learning enhanced my self-study ability	90(79.65)
I gained less from fully online learning than from classroom teacher-led learning	23(20.35)
I did not learn anything from the pure online course.	0(0)
*What do you think of pure online learning?*	
Excellent	44(38.94)
Good	62(54.87)
Average	7(6.19)

## 4 Discussion

The Shandong First Medical University, China introduced blended learning for dermatology courses in 2019, receiving positive feedback from students. As part of the online component of the blended course, high-quality SPOCs were designed. Students first studied the SPOC videos and then attended face-to-face classes with teachers to clarify their questions and doubts.

The outbreak of the COVID-19 pandemic prompted a reform in blended teaching as students had to study at home due to lockdown regulations. In such circumstances, face-to-face classroom teacher-led learning was transitioned to live teacher-led learning, ensuring the continuity of the learning process. Several studies have compared traditional classroom teaching and online teaching and analyzed their effects on student efficiency (8–11). However, there is limited research on the distinctions between traditional blended teaching and fully online teaching. The present study compared the average scores of undergraduate students in dermatology courses at the Clinical Medical College for the academic years 2019 and 2020, when traditional blended learning and fully online teaching were introduced, respectively. The researchers also analyzed students’ responses to a questionnaire survey conducted in 2020 to evaluate their perspectives on the two learning modes.

The findings revealed that students who took pure online courses performed less effectively than those who participated in traditional blended learning. However, there were no differences in the distribution of achievement variance at different levels between the two groups. Poor student engagement and a lack of self-discipline might contribute to lower academic performance in pure online learning. During face-to-face teaching, students need to be present in the classroom, actively listen to the teacher, and participate in class. In contrast, during live teacher-led learning, students must be disciplined and motivated to avoid distractions without any intervention from the teachers. Students face greater challenges during pure online learning, especially in terms of time management and learning persistence (12).

Educators also encounter certain challenges that contribute to the lower effectiveness of pure online learning. First, educators become frustrated and emotionally exhausted when students remain absent from online courses. Second, teachers can only see profile photos of students during online classes and cannot assess whether students have grasped the content, making it difficult to engage with inactive students. Third, students need hands-on experience to observe, touch, and smell various lesions to understand skin diseases, but this is impossible to achieve during online clerkship courses. Consequently, more focused lessons are required for online learning. Svoboda et al. found that “high-yielding” and more interactive lectures can be created by focusing on higher-order concepts that challenge learners to apply and synthesize clinical data. They advised educators to teach concepts and ask questions that cannot be readily answered through search engines. In this way, students can learn the fundamental facts independently and combine their knowledge with critical thinking, maximizing learning efficiency and increasing engagement (13). Furthermore, advanced technology is needed to enhance online dermatology education. For instance, virtual reality has the potential to enrich learning by providing students with access to virtual environments where they can interact with immersive content from various subjects.

Interaction is another crucial factor influencing the effectiveness of online learning. Prior studies have shown that students’ interaction significantly impacts their learning persistence, which is essential in online learning (12). Caldwell (14) found that high levels of interaction between students and their environments (peers, instructors, and content) can create an engaging and enjoyable learning atmosphere. Yu et al. (15) discovered that academic emotions play the role of both a moderator and a mediator in the relationship between students’ interaction and learning persistence in online learning environments. De Felice and colleagues (16) observed that social richness also has an impact on learning. For example, communication cues like gestures of a teacher can enhance learning by directing visual attention and synchronizing with speech (17). De Felice et al. concluded that live teaching during online sessions has a more significant positive impact on learning compared to pre-recorded teaching, reaffirming the crucial role of interaction in online learning (15, 16, 18). Therefore, educators should aim to improve students’ moods and create an enjoyable atmosphere through increased interactions to enhance their learning outcomes, while limiting the use of pre-recorded learning hours. Tanaka et al. found that daily 15-min interactive lectures based on a single anesthesiology keyword and a case scenario with board-style questions could improve resident post-rotation evaluation scores and increase engagement (19). King et al. argued that a “flipped classroom” as an interactive lecture mode might create a more relaxed learning atmosphere for students by eliminating the perceived risk of personal failure or embarrassment that exists in traditional teaching modes (20).

The analysis of the questionnaire responses revealed that, like all other modes of teaching, online education has its advantages and disadvantages. More than half of the respondents identified a flexible schedule, repeated access to learning content, and a relaxed environment as significant advantages of online learning. Being overly reliant on a stable network connection, low learning motivation, and limited social interactions were the major disadvantages associated with online learning, mirroring the findings of previous studies (21, 22). Furthermore, students preferred interaction during or immediately after teacher-led learning through a live meeting application over SPOC discussion forums or discussions on WeChat, reinforcing the importance of live interactions during online learning. When it comes to online teaching methods, most students favored a combination of online video self-study and live teacher-led learning over a single mode of video self-study or live teacher-led learning. Regarding learning methods, most students preferred offline clerkship courses over online courses and offline classroom teaching. Dermatology is characterized by visible symptoms, and direct patient contact is significant for undergraduate students because it can stimulate visual impressions and motivation. Therefore, live clerkship courses need to be well-designed to simulate real-life scenarios. Once such a system is in place, the effectiveness of live clerkship courses will significantly improve. Additionally, students hoped that teachers could provide various learning materials such as course PowerPoint presentations, videos, online homework, and e-textbooks, and suggest related websites or books, and related literature. However, it was observed that many students primarily use these materials to pass the final exam, rather than gaining a deeper understanding of skin diseases.

Despite the aforementioned limitations of the fully online teaching mode, the majority of students in 2020 demonstrated a solid command of “dermatology” concepts and successfully passed the exam. Most students indicated that pure online learning enhanced their self-study ability, a crucial skill for medical students in the Internet age, aligning with our educational goals. Given that lifelong learning is a critical skill for health professionals, the educational focus must shift from knowledge acquisition to the development of self-study ability. The fully online teaching mode in 2020 received particularly high ratings in terms of “student satisfaction and learning effectiveness,” with an overwhelming majority rating the course as excellent or good.

The findings of this study should be considered in light of certain limitations. Firstly, the sudden transition to fully online learning during the COVID-19 pandemic in 2020 did not give the students any time to adjust to the transition. Therefore, the results of the present study may not fully reflect the real effect of purely online courses. Secondly, the study focused on the dermatology department of a single university, which introduces bias into the sampling profile and affects the generalizability of the findings. Thirdly, the study adopts a retrospective design, making it susceptible to biases. A prospective multi-center study involving students from various majors should be conducted in the future to provide new insights into optimizing online learning.

## 5 Conclusion

This study shows that fully online learning is currently not as effective as traditional blended learning due to some limitations. However, online learning is rapidly replacing traditional learning methods, and students also believe that pure online learning can enhance their self-study ability. Therefore, online courses should be improved to ignite students’ interest and increase their learning efficiency, preparing medical students for clinical work in the near future.

## Data availability statement

The raw data supporting the conclusions of this article will be made available by the authors, without undue reservation.

## Ethics statement

The studies involving humans were approved by Biomedical Research Ethical Committee of Shandong Provincial Hospital. The studies were conducted in accordance with the local legislation and institutional requirements. Written informed consent for participation was not required from the participants or the participants’ legal guardians/next of kin in accordance with the national legislation and institutional requirements.

## Author contributions

ND conceived and designed the experiments. YM, ND, and JJ performed the experiments. YM, MS, JG, and ND analyzed the data. MS and JG contributed materials/analysis tools. YM wrote the paper. ND edited the paper. All authors contributed to the article and approved the submitted version.

## Footnotes

^1^https://www.icourse163.org/spoc/course/SDU-1205724812
